# Incidence and outcome of BCR‐ABL mutated chronic myeloid leukemia patients who failed to tyrosine kinase inhibitors

**DOI:** 10.1002/cam4.2410

**Published:** 2019-07-27

**Authors:** Gabriel Etienne, Stéphanie Dulucq, Françoise Huguet, Anna Schmitt, Axelle Lascaux, Sandrine Hayette, Marie‐Pierre Fort, Pierre Sujobert, Fontanet Bijou, Stéphane Morisset, Suzanne Tavitian, Audrey Bidet, Beatrice Turcq, Fanny Robbesyn, Claudine Chollet, Francis Belloc, Françoise Durrieu, François‐Xavier Mahon, Franck E. Nicolini

**Affiliations:** ^1^ Département d'Hématologie Institut Bergonié Bordeaux France; ^2^ Laboratory of Mammary and Leukaemic Oncogenesis INSERM U1218, Université de Bordeaux Bordeaux France; ^3^ Groupe Fi‐LMC, Hôpital Haut‐Lévêque Pessac France; ^4^ Laboratoire d'Hématologie Hôpital Haut Lévêque CHU de Bordeaux Pessac France; ^5^ Service d'Hématologie Institut Universitaire du Cancer Toulouse‐Oncopole, Centre Hospitalier Universitaire Toulouse France; ^6^ Service des maladies du sang Hôpital Haut Lévêque CHU de Bordeaux Pessac France; ^7^ Laboratoire d'Hématologie Centre Hospitalier Lyon Sud Pierre Bénite France; ^8^ Hematology Department Centre Léon Bérard Lyon France; ^9^ Service d'Hématologie et INSERM U 1052, CRCL, Centre Léon Bérard Lyon France; ^10^ INSERM U1052, CRCL, Centre Léon Bérard Lyon France

**Keywords:** BCR-ABL kinase domain mutation, chronic myeloid leukemia, tyrosine kinase inhibitors

## Abstract

**Purpose:**

To assess the incidence of BCR‐ABL kinase domain (KD) mutation detection and its prognostic significance in chronic phase chronic myeloid leukemia (CP‐CML) patients treated with tyrosine kinase inhibitors (TKIs).

**Patients and Methods:**

We analyzed characteristics and outcome of 253 CP‐CML patients who had at least one mutation analysis performed using direct sequencing. Of them, 187 patients were early CP (ECP) and 66 were late CP late chronic phase (LCP) and 88% were treated with Imatinib as first‐line TKI.

**Results:**

Overall, 80 (32%) patients harbored BCR‐ABL KD mutations. A BCR‐ABL KD mutation was identified in 57% of patients, who progressed to accelerated or blastic phases (AP‐BP), and 47%, 29%, 35%, 16% and 26% in patients in CP‐CML at the time of mutation analysis who lost a complete hematologic response, failed to achieve or loss of a prior complete cytogenetic and major molecular response, respectively.

Overall survival and cumulative incidence of CML‐related death were significantly correlated with the disease phase whatever the absence or presence of a mutation was and for the latter the mutation subgroup (T315I vs P‐loop vs non‐T315I non‐P‐loop) (*P*<.001). Considering patients who were in CP at the time of mutation analysis, LCP mutated patients had a significantly worse outcome than ECP‐mutated patients despite a lower incidence of T315I and P‐loop mutations (*P*<.001). With a median follow‐up from mutation analysis to last follow‐up of 5 years, T315I and P‐loop mutations were not associated with a worse outcome in ECP patients (*P *= .817).

**Conclusion:**

Our results suggest that early mutation detection together with accessibility to 2nd and 3rd generation TKIs have reversed the worst outcome associated with BCR‐ABL KD mutations whatever the mutation subgroup in CP‐CML patients.

## 1 INTRODUCTION

Imatinib (IMA) has significantly improved the prognosis of chronic myeloid leukemia (CML) with life expectancy for CML patients now close to the healthy population.^1^, ^2^, ^3^ However, a subset of patients develops IMA resistance. Several possible mechanisms of resistance have been demonstrated in vitro. The occurrence of BCR‐ABL kinase domain (KD) mutations remains the most documented one in vivo, identified in at least 50% of tyrosine kinase inhibitors (TKIs) failures and disease progression.^4^, ^5^, ^6^ To date, more than 100 emergent mutations have been reported in association with various degrees of resistance to IMA.^7^ Among these, T315I and P‐loop mutations have been associated with the highest level of IMA resistance and a worst clinical outcome as compared to other mutations.^8^, ^9^, ^10^, ^11^, ^12^, ^13^ Based on preclinical studies, the level of resistance to 2nd and 3rd generation TKIs (2‐3GTKIs) has been determined for a large panel of mutations allowing a rational use of TKIs in IMA failure patients.^11^, ^12^, ^13^ Whether accessibility to 2‐3GTKIs together with adequate TKI management according to the type of mutation have improved the outcome of CML patients harboring a BCR‐ABL KD mutation in a TKI failure situation remains to be determined. The aims of this retrospective study were to determine the incidence of BCR‐ABL KD mutations according to patient's baseline characteristics and TKI failure situations at the time of mutation analysis (using Sanger sequencing) and to analyze the outcome of CML patients with and without mutations.

## 2 PATIENTS AND METHODS

### 2.1 Patients

Patients with a Philadelphia chromosome‐positive CML in chronic phase at diagnosis (CP‐CML) who had at least one BCR‐ABL KD mutation analysis on peripheral blood using Sanger sequencing in the 3 participating French centers were included in this study. Mutation analyses were required by physicians in patients who have unsatisfactory responses to TKIs. Baseline characteristics (age, gender, Sokal score) together with ongoing characteristics (phase of the disease, disease status, treatment and response) at the time of mutation analysis and clinical outcome were defined by conventional criteria.^14^, ^15^ All patients provided informed consent to participate in the study. This study has been approved by the local Ethics Committee and the French Data Protection Authority (Commission Nationale de l'Informatique et des Libertés, CNIL).

### 2.2 Study evaluations

Overall survival (OS) was measured from the date of the mutation analysis to the date of last follow‐up or death. For patients who underwent allogenic hematopoietic stem cell transplantation (AHSCT), follow‐up was censored at the AHSCT date. For patients who had more than one mutation screen, the date of the first positive assessment for BCR‐ABL KD mutation was retained. In the absence of BCR‐ABL KD mutation, the date of the first negative mutation analysis was considered. To assess the prognostic impact of BCR‐ABL KD mutations, patients were analyzed according to the type of the first identified mutation and classified into three mutation subgroups, ie T315I, P‐loop (amino acid substitution between residues 248 and 255) and non‐T315I non‐P‐loop. When more than one mutation was detected in a patient sample the predominant clone was only considered. The BCR‐ABL mutation detection was performed as previously described.^9^

### 2.3 Statistical methods

The patient characteristics were analyzed through descriptive statistics. Bivariate analysis according to the mutational state were completed by the appropriated statistical test such as Mann‐Whitney (with exact approximation if needed) or Student's *t* test for continuous variables, and Pearson's Chi‐square or Monte‐Carlo tests for qualitative variables.

Overall survival since the date of mutation analysis was illustrated with the Kaplan‐Meier method, along with a log‐rank test. Death events associated with CML from the date of mutation analysis (with death from any other cause as competing risk) were represented with incidence function, along with a Gray test for curve comparisons. The level of significance was set at 5%, the estimation of hazard ratios was described with an interval of confidence of 95%. All the statistical analyses were executed using the 'survival', 'cmprsk' and 'ggplot2' packages of the R program (version 3.2.3).

## 3 RESULTS

### 3.1 Study population

From February 2002 to March 2016, 253 CML patients in CP at diagnosis underwent at least one BCR‐ABL mutation screening using Sanger sequencing. The baseline characteristics of the study population together with disease status and treatment at the time of mutation analysis are presented in Table 1. Of the 253 patients, 66 patients had been previously treated with interferon‐based regimens before TKI start (considered as late chronic phase [LCP]) and 187 (73.9%) were treated with TKI front‐line and defined as early chronic phase (ECP). The median time from diagnosis to TKI start for LCP patients was 4.59 years (range 0.15‐26.73). IMA was the first‐line TKI in 87.7% of the cases. At the date of the mutation analysis 211 (83.4%) patients were in CP and 190 (75.1%) patients were treated with IMA.

**Table 1:** Characteristics of the study population (n = 253 patients) with (n = 80 patients) and without (n = 173 patients) BCR‐ABL tyrosine kinase domain mutation determined by direct sequencing at baseline and at the time of mutation analysis

Characteristics	Patients (N = 253)	Patients with BCR‐ABL KD mutation (N = 80)	Patients without BCR‐ABL KD mutation (N = 173)	*P*‐value
Median age at diagnosis, y (range)	52 (17‐82)	54 (19‐80)	51 (17‐82)	.26
Gender, female, n pts (%)	108 (42.7)	33 (41.2)	75 (43.4)	.859
Sokal risk score, n pts (%)				
Low	59 (23.3)	12 (15.0)	47 (27.2)	.049
Intermediate	77 (30.4)	23 (28.7)	54 (31.2)	
High	86 (34.0)	36 (45.0)	50 (28.9)	
Unknown	31 (12.3)	9 (11.2)	22 (12.7)	
Status at TKI start				
Newly diagnosed CP pts, n (%)	187 (73.9)	47 (58.8)	140 (80.9)	<.001
CP pts previously treated with IFNα, n, (%)	66 (26.1)	33 (41.2)	33 (19.1)	
Median time from diagnosis to TKI start, yrs (range)^a^	4.59 (0.15‐26.73)	4.6 (0.17‐23.72)	4.58 (0.15‐26.73)	.26
First‐lineTKI type, n pts (%)				
Imatinib	222 (87.7)	71 (88.8)	151 (87.3)	.26
Nilotinib	17 (6.7)	7 (8.8)	10 (5.8)	
Dasatinib	14 (5.5)	2 (2.5)	12 (6.9)	
Median time from first‐line TKI start to mutation analysis, yrs (range)	2.5 (0.02‐11.26)	3.38 (0.25‐9.72)	2.02 (0.02‐11.26)	.005
CML Phase at the time of mutation analysis, n pts (%)				
Chronic Phase	211 (83.4)	56 (70.0)	155 (89.6)	<.001
Accelerated Phase	23 (9.1)	12 (15.0)	11 (6.4)	
Blastic Phase	19 (7.5)	12 (15.0)	7 (4.0)	
TKI type at the date of mutation analysis, n pts (%)				
Imatinib	190 (75.1)	54 (67.5)	136 (78.6)	.017
Nilotinib	36 (14.2)	17 (21.2)	19 (11.0)	
Dasatinib	25 (9.9)	7 (8.8)	18 (10.4)	
Ponatinib	2 (0.8)	2 (2.5)	0 (0)	

Abbreviations: IFNα, interferon α; KD, kinase domain; pts, patients; TKI, tyrosine kinase inhibitor.

a For late chronic phase patients.

At least one BCR‐ABL KD mutation was identified in 80 of them with a median time from TKI start to mutation identification of 3.38 years (range, 0.25‐9.72). Four patients harbored more than one mutation at the time of the analysis (T315I + E255K in one patient, included in the T315I subgroup; E255K + T315I and Y253H + E255K in one patient each, both included in the P‐loop subgroup; M244V + E279K in one patient, included in the non‐T315I non‐P‐loop subgroup).

**Figure 1:**
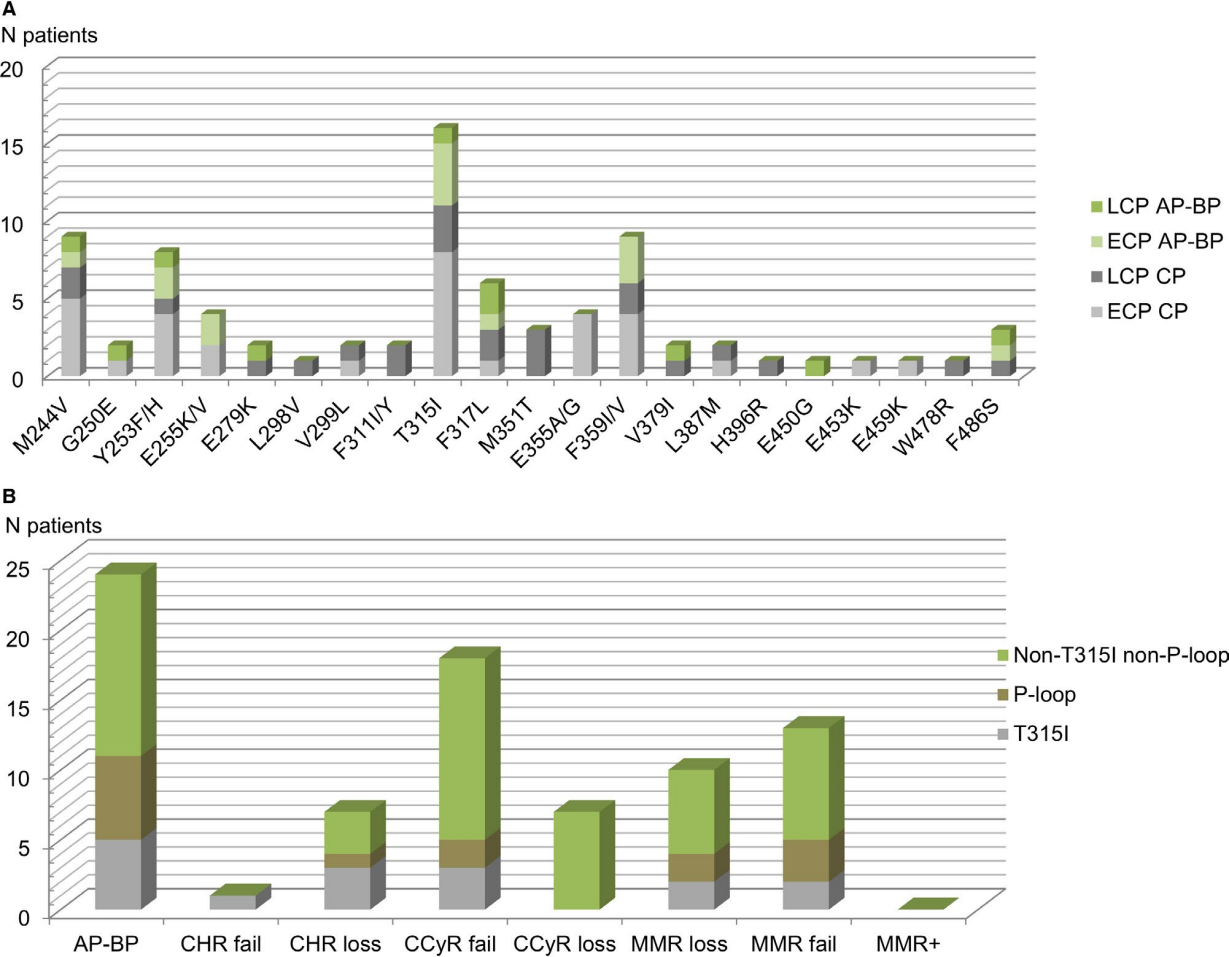
BCR‐ABL tyrosine kinase domain mutations (n = 80 patients) according to status at TKI start (early chronic phase (ECP) vs late chronic phase (LCP)) disease phase (chronic phase (CP) vs accelerated and blastic phases (AP‐BP)) at the time of mutation analysis (1A); mutation subgroups patients according to disease status (AP‐BP; failure to achieve or loss of complete hematologic response (CHR); failure to achieve or loss of complete cytogenetic response (CCyR); failure to achieve or loss of major molecular response (MMR)) at the time of mutation analysis; sustained MMR with BCR‐ABL increase of one log (1B)

The type of mutation according to the status at TKI start (LCP vs ECP) and the phase of the disease at the time of mutation analysis together with the mutation subgroups according to the reason for mutation screen are presented in Figure 1.

### 3.2 Baseline and ongoing characteristics associated with mutation detection

Baseline characteristics associated with a significant probability of a positive mutation analysis were high Sokal risk score (*P *= .049) and LCP status (*P* < .001). Rates of positive and negative mutation analysis as well as median time from first TKI start to mutation occurrence, according to the status disease at the time of mutation screen are presented in Table 2.

**Table 2:** Median time from first‐line TKIs start and to presence (N = 80 patients) or absence (N = 173 patients) of BCR‐ABL KD mutations according to disease status at the time of mutation analysis

Disease status at the time of mutation analysis	N pts	Median time from TKI start to mutation analysis, y (range)	Positive mutation analysis N = 80	Negative mutation analysis N = 173
Progression to AP or BP, n pts (%)	42	3.35 (0.12‐8.78)	24 (57.1)	18 (42.8)
Failure to achieve or loss of CHR, n pts (%)	17	2.84 (0.25‐9.72)	8 (47.0)	9 (53.0)
Failure to achieve CCyR, n pts (%)	61	1.14 (0.04‐11.27)	18 (29.5)	43 (70.4)
Loss of CCyR, n pts (%)	20	2.62 (0.27‐7.87)	7 (35.0)	13 (65.0)
Failure to achieve MMR, n pts (%)	53	1.64 (0.73‐9.31)	9 (16.9)	44 (83.0)
MMR loss, n pts (%)	53	5.01 (0.99‐11.05)	14 (26.4)	39 (73.5)
Sustained MMR with BCR‐ABL transcript increase, n pts (%)	7	3.0 (0.23‐9.30)	0 (0)	7 (100)

Abbreviations: AP, accelerated phase; BP, blastic phase; CCyR, complete cytogenetic response; CHR, complete hematologic response; KD, kinase domain; MMR, major molecular response; pts, patients; TKI, tyrosine kinase inhibitor.

### 3.3 Outcome

The median follow‐up durations (n = 253 patients) from diagnosis and mutation analysis to the date of last follow‐up or death were 8.18 years (range 0.84‐32.73) and 3.32 years (range 0.0 to 13.54), respectively. Overall 52 patients (20.5%) died and death was related to CML in 35 (13.8%) of them. Among the 211 patients who were in CP at the time of mutation analysis, 23 have died (12 in the mutated group with 8 CML‐related deaths and 11 in the non‐mutated group with 3 CML‐related deaths). During follow‐up, 14 patients (11 patients without mutation and 3 patients with positive mutation detection: M244V, T315I and E355G) who were in CP at the date of mutation analysis and 15 patients in AP or BP at the time of mutation analysis underwent AHSCT.

### 3.4 Outcome according to disease phase at the time of mutation analysis, status at TKIs initiation and mutational results

The outcome was analyzed according to the disease phase at the time of mutation analysis (CP vs AP‐BP), to the status at the time of TKI initiation (LCP vs ECP), to the presence or absence of mutation and for the latter to the mutation subgroup (T315I vs P‐loop vs non‐T315I non‐P‐loop).

### 3.5 **Outcome according to disease phase at the time of mutation analysis (CP **vs** AP‐BP) and mutational status (presence or absence of a BCR‐ABL KD mutation; n = 253 pts)**

**Figure 2:**
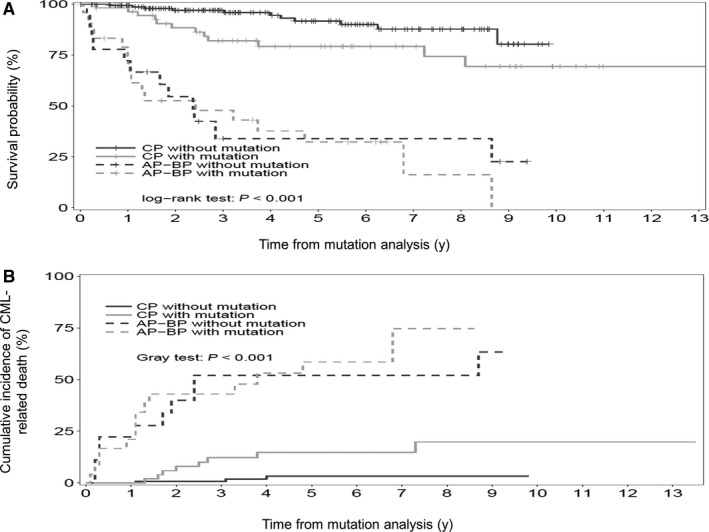
Overall survival (2A) and cumulative incidence of CML‐related death (2B) from the time of mutation analysis to the last follow‐up according to the phase of the disease at the time of mutation analysis (chronic phase, CP; accelerated and blastic phase, AP‐BP) and to the absence or presence of a BCR‐ABL tyrosine kinase domain mutation (n = 253 patients; CP without mutation, n = 155 pts; CP with mutation, n = 56 pts; AP‐BP without mutation, n = 18 pts; AP‐BP with mutation, n = 24 pts).The probability of OS and rates of CI of CML‐related death at 5 y were 91% (95% CI: 86‐97.9) and 3.2% (95% CI: 0‐6.9), 79% (95% CI: 68.5‐91.7) and 14.8% (95% CI: 4.4‐25.1), 33.9% (95% CI: 16.7‐68.7) and 52% (95% CI: 27‐76.9), 32% (95% CI: 17.3‐60.1) and 58% (95% CI: 36.4‐80.6) for CP without mutation, CP with mutation, AP‐BP without mutation and AP‐BP with mutation, respectively (OS, *P *< .001; CI CML‐related deaths, *P *< .001)

Overall survival and rate of cumulative incidence (CI) of CML‐related death were significantly correlated with the disease phase at the time of mutation analysis (CP vs AP‐BP) whatever the presence or absence of a BCR‐ABL KD mutation (Figure 2). Of note, OS of the 42 patients in AP or BP at the time of mutation analysis did not differ when the subgroup of mutation was taken into account (data not shown).

### 3.6 Outcome according to status at the time of TKI initiation (LCP vs ECP) a and mutational status (presence or absence of a BCR‐ABL KD mutation) in patients in CP at the date of mutation analysis (n = 211 pts)

We next analyzed the outcome of patients in CP at the date of mutation analysis (n = 211 patients) according to the patient's status at the time of first‐line TKIs initiation (ECP vs LCP). A BCR‐ABL KD mutation was identified in 56 patients (LCP, 23 patients; ECP, 33 patients; detailed in Figure 1).

**Figure 3:**
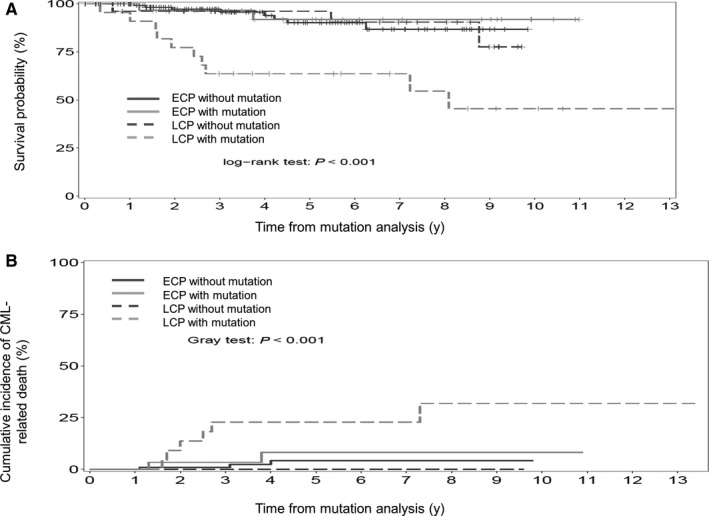
Overall survival (3A) and cumulative incidence of CML‐related death (3B) from the time of mutation analysis to the last follow‐up according to status at TKI start ((early chronic phase (ECP) vs late chronic phase (LCP)) and absence or presence of a BCR‐ABL tyrosine kinase domain mutation (n = 211 patients; ECP without mutation, n = 128 pts; ECP with mutation, n = 33 pts; LCP without mutation, n = 27 pts; LCP with mutation, n = 23 pts).The probability of OS and rates of CI CML‐related death at 5 y were 90% (95% CI: 83.1‐97.7) and 4% (95% CI: 0‐8.8), 91% (95% CI: 81.4‐100) and 8% (95% CI: 0‐19.4), 96% (95% CI: 89‐100) and 0% (95% CI: 0‐0), 64% (95% CI: 46.4‐87.7) and 23% (95% CI: 4.7‐40.7) for ECP without mutation, ECP with mutation, LCP without mutation and LCP with mutation, respectively (OS, *P*<.001; CI CML‐related deaths, *P*<.001)

Late chronic phase mutated patients had a significantly poor outcome while ECP patients with and without mutation and non‐mutated LCP patients demonstrated no significant difference in OS and CI of CML‐related deaths (Figure 3).

Ongoing characteristics of LCP and ECP patients who harbored a BCR‐ABL KD mutation were then compared. Median time from TKI start to the positive mutation analysis in LCP and ECP patients were 2.7 and 4 years, respectively. At the time of mutation analysis, 30%, 43% and 26% of LCP mutated patients and 6% 45% and 48% of ECP mutated patients were in hematologic, cytogenetic and molecular failures, respectively. Irrespective of the mutation subgroup, OS and CI CML‐related death appeared significantly correlated with the type of failure. Indeed, patients with hematologic failure had the worst outcome and those with molecular failure patients the best, while those with cytogenetic failure patient had intermediate outcome (data not shown). Among the 56 mutated patients, 49 underwent a TKI change following mutation detection within a median time of 0.55 year (range, 0.0‐3.24) and 0.26 year (range, 0.0‐5.14) in LCP and ECP patients. Seven patients did not have any therapeutic change following mutation detection. Among them, mutation analysis was positive at the date of last follow‐up in one and one patient harboring a M244V was still on IMA with a sustained complete cytogenetic response (CCyR). The remaining five patients (all LCP) had no available alternative therapeutic following mutation identification. Four of them died due to CML progression in 3 and concomitant neoplasia in one.

### 3.7 Outcome according to the mutational status (absence vs presence) and subgroup mutation (T315 vs P‐loop vs non‐T315 non P‐loop) in patients treated with TKI‐frontline and in CP at the date of the mutational analysis (n = 161 pts)

Finally, we analyzed the outcome of patients who were treated with de novo TKI (n = 161 patients) according to the absence (n = 128 patients) or the presence (n = 33 patients) of a mutation and to the subgroup of mutation (T315I, 8 patients; P‐loop, 7 patients and non‐T315I non‐P‐loop, 18 patients). Median times from first‐line TKI start to mutation detection and from mutation detection to last follow‐up were 2.12 years (range, 1.08‐6.31) and 5.19 years (range, 1.52‐8.73) in the T315I subgroup, 2.78 years (range, 0.99‐5.19) and 4.4 years (range, 0.27‐9.93) in the P‐loop subgroup and 3.15 years (range, 0.69‐6.79) and 5.44 years (rage, 1.0‐10.99) in the non‐315I non‐P‐loop subgroup, respectively. With the exception of two patients who died from CML progression (one in the T315I subgroup and one in the non‐T315I non‐P‐loop subgroup), all patients were alive at last follow‐up. Two patients underwent AHSCT (one in the T315I subgroup, one in the non‐T315I non‐P‐loop subgroup) and were in undetectable molecular residual disease at the time of last follow‐up without TKI. TKI treatments at the date of mutational analysis and at the date of last follow‐up of the 33 mutated patients are summarize in Table 3.

**Table 3:** Tyrosine kinase inhibitor treatments at the date of mutational analysis and at the date of last follow‐up of patients treated with frontline TKI, in CP at the date of mutational analysis and habored a BCR‐ABL KD mutation (n = 33 patients)

	T315I N = 8 pts	P‐loop N = 7 pts	Non‐T315I non‐P‐loop N = 18 pts
TKI at the date of mutational analysis, n pts			
IMA	4	4	13
DAS	3	0	4
NIL	1	3	1
TKI at the date of last follow‐up, n pts			
None	2^a^	0	0
IMA	0	0	1
NIL	0	1	7
DAS	0	4	7
PON	6	2	3

Abbreviations: DAS, dasatinib; IMA, imatinib; NIL, nilotinib; PON, ponatinib; TKI, tyrosine kinase inhibitor.

a One patient in undectable molecular residual disease after allogenic hematopoietic stem cell transplantation, one patient treated with omacetaxine.

**Figure 4:**
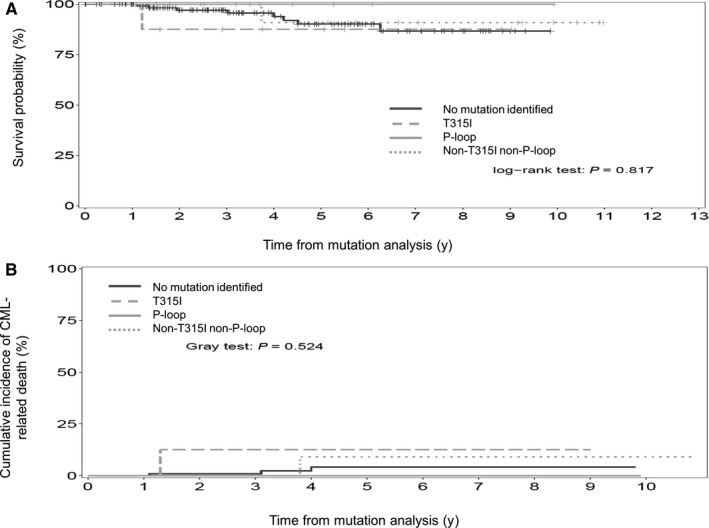
Overall survival (4A) and cumulative incidence of CML‐related death (4B) of early chronic phase patients (ECP) (n = 161 patients) from the time of mutation analysis to the last follow‐up according to absence of mutation (n = 128 patients) or presence and to type of mutation (n = 33 patients; T315I mutation, n = 8 pts; P‐loop mutation, n = 7 pts; non‐T315I non‐P‐loop mutations, n = 18 pts). Indeed, the probability of OS and rates of CI CML‐related deaths at 5 y were 91% (95% CI: 86‐97.6) and 4% (95% CI: 0‐8.8), 71% (95% CI: 48.8‐100) and 12% (95% CI: 0‐37), 100% (95% CI: 100‐100) and 0% (95% CI: 0‐0), 79% (95% CI: 66.3‐94.3) and 9% (95% CI: 0‐26.9) for ECP without mutation, ECP with T315I mutation, ECP with P‐loop mutation and ECP with non‐T315I non‐P‐loop mutation, respectively (OS, *P *= .817; CI CML‐related deaths, *P *= 0.524)

Overall survival and CI of CML‐related deaths were not statistically different between non‐mutated and mutated patients whatever the type of mutation (Figure 4). Two patients who were initially classified in the non‐T315 non‐P‐loop subgroup (M244V, one patient; E355A, one patient) have subsequently developed a P‐loop mutation (E255K) while still in CP. Then, the prognostic impact of mutation subtype was analyzed according to the presence of a P‐loop mutation at any time as long as the patients were still in CP at the date of mutation analysis. Again, no significant prognostic impact was observed (data not shown).

## 4 DISCUSSION

The objectives of this study were to assess the survival of CP‐CML patients treated with TKI in a daily clinical practice together with baseline and ongoing patients’ characteristics associated with a positive BCR‐ABL KD mutation detection using Sanger sequencing. Most of the patients were treated with frontline IMA and in TKI‐failure situations at the date of mutation screening. Altogether, a BCR‐ABL KD mutation was identified in 31% of the patients and in 25% when considering patients in CP only at the time of mutation analysis.

Mutation analysis is currently recommended in case of TKI failure.^7^, ^14^ Irrespective of TKI discontinuation related to drug intolerance, TKI failure in CP patients covers different clinical situations summarized as progression to advanced disease phases, failure to achieve response at defined milestones and loss of previous response according to hematologic, cytogenetic or molecular criteria.^14^, ^16^ Preliminary reports have pointed out that the emergence of mutations in CP patients preferentially occurs during disease progression to AP‐BP and in secondary rather than primary IMA‐resistance usually defined according to cytogenetic criteria.^6^, ^17^

In this setting, median time from TKI start to mutation detection according to the disease status at the time of mutation search and the corresponding mutation incidence are informative. Indeed, in this study patients who failed to achieve or lost a previous CCyR have a median time from TKI start to mutation analysis of 1.1 and 2.6 years, respectively, consistent with the cytogenetic failure event calendar previously reported in de novo IMA‐treated CP‐CML patients.^18^, ^19^ The corresponding mutation incidences are very similar (29% in CCyR failure vs 35% if CCyR loss) emphasizing the fact that mutation search should be performed irrespective of the cytogenetic resistance profile and as recommended.^7^

Considering patients who underwent mutation screening due to failure of achieving major molecular response (MMR) or loss of previous MMR, the mutation incidences were 16.9% and 26%, respectively.

Median time from TKI start to mutation analysis was 1.6 year in patients who failed to achieve MMR highlighting the fact that patients in suboptimal response according to the 2009 European LeukemiaNet proposed criteria for TKI response evaluation eventually may harbor BCR‐ABL KD mutations.^16^ Interestingly, the median time from TKI start to mutation analysis in patients who have MMR loss was 5 years (4.7 years in case of negative result, 5 years in case of positive result) illustrating that mutation emergence associated with MMR loss may be a late event.

The second objective of this study was to assess the prognostic impact of BCR‐ABL KD mutation. Consistent with previous studies, the disease phase at the time of mutation analysis is associated with a poorer outcome whatever the absence or presence of a mutation and for the latter the mutation subgroup.^20^, ^21^, ^22^, ^23^ Although, therapeutic management of AP‐BP CML patients remains heterogeneous and mainly depends on the feasibility of TKI switch with or without high dose chemotherapy followed by AHSCT, the presence of a BCR‐ABL KD mutation is not associated with a worse outcome whatever the subgroup of mutation suggesting that BCR‐ABL KD mutations in late disease phases are one but not the main leukemic evolution driver. Moreover, irrespective of mutational status, none of the currently available TKIs has demonstrated a survival advantage in BP as recently demonstrated by Jain et al^24^

We next analyzed the outcome of patients in CP at the date of mutation analysis according to patient status at the date of TKI start (LCP vs ECP). As already reported in previous studies, LCP patients with BCR‐ABL KD mutations have a poor outcome and, in this study the worst when compared to other groups.^8^, ^13^ Several explanations may be suggested. On the first hand, median time from TKI start to mutation analysis was longer in the LCP group when compared to ECP (4 vs 2.7 years). On the second hand, the proportion of patients who were in hematologic, cytogenetic and molecular failures in both groups were different with a higher proportion of patients in hematologic failure and a lower incidence of molecular failure in LCP patients.

In spite of a lower incidence of T315I and P‐loop mutations among LCP patients when compared to ECP (Figure 1) and a median time from mutation analysis to therapeutic change following mutation analysis closed to the ECP group (0.55 vs 0.26 year), LCP mutated patients have a worst outcome.

Taken together, our results suggest that detection of BCR‐ABL KD mutation in hematologic failure and lack of alternative therapies following mutation detection may account for the worse outcome of LCP mutated patients when compared to others. T315I and P‐loop mutations have been associated in CP patients with a significant worse outcome several years ago.^6^, ^9^, ^10^

However, many patients reported in the corresponding published studies were LCP without for many of them immediate accessibility to 2‐3GTKIs following mutation identification. With a median follow‐up of 5 years after mutation detection, the negative prognostic impact of T315I and P‐loop mutation was not observed in this study. These results suggest that earlier detection of BCR‐ABL KD mutation together with accessibility to 2‐3GTKI and therapeutic change based on preclinical and clinical available data on TKI sensitivity for a given mutation have significantly improved the outcome of mutated patients, as long as the disease remains in CP at the time of mutation identification. Previous published studies have demonstrated that, using more sensitive techniques such as next generation sequencing (NGS), BCR‐ABL KD mutations can be detected earlier and at a very low level.^25^, ^26^ Whether early mutation detection using NGS together with the switch to other currently available TKIs will prevent progression of the disease in mutated patients is currently under investigation. However, our study already points out that early detection of BCR‐ABL mutation with conventional techniques and accessibility to 2‐3GTKIs have currently reversed the worse outcome of T315I and P‐loop mutated CP‐CML patients.

## CONFLICTS OF INTEREST

None declared.
